# Cellulose-Based Nanosponges with PEDOT:PSS as Pressure Sensors with Tunable Elasticity: A Study on Modulation of Mechanical Properties and Pressure Response

**DOI:** 10.1021/acsomega.6c03688

**Published:** 2026-10-08

**Authors:** V. Vit, L. Riva, V. Sinisi, A. Fiorati, A. Alam, A. B. Alabi, C. Punta, N. Coppedè

**Affiliations:** † Institute of Materials for Electronics and Magnetism (IMEM), National Research Council (CNR), Parco Area Delle Scienze 37/A, 43124 Parma, Italy; ‡ Department of Chemistry, Materials, and Chemical Engineering “G. Natta” Politecnico di Milano and INSTM Local Unit, Piazza Leonardo Da Vinci 32, I-20133 Milano, Italy; § University of Parma, Italy, Department of Chemistry, Life Sciences and Environmental Sustainability, Parco Area Delle Scienze, 17/A, 43124 Parma, Italy

## Abstract

This study investigates the suitability of sustainable, highly porous nanosponges, composed of TEMPO-oxidized cellulose nanofibers (TOCNF) and branched polyethylenimine (bPEI, cross-linker), as scaffolds for PEDOT:PSS poly­(3,4-ethylenedioxythiophene):polystyrenesulfonate dip coating to design piezoresistive pressure sensors. The mechanical properties of the nanosponges were tuned by introducing citric acid (CA) as a co-cross-linker, varying its content from 0% to 18% relative to the moles of primary amines of bPEI. The resulting devices functioned effectively as pressure sensors, with the best performance achieved at 3% CA. This configuration operated reliably up to 200 kPa and reached a sensitivity of 1.96 × 10^–3^ mA·kPa^–1^. These findings confirm the promising performance of cellulose-based nanosponges and pave the way for their integration into lightweight and environmentally friendly pressure sensors.

## Graphical abstract

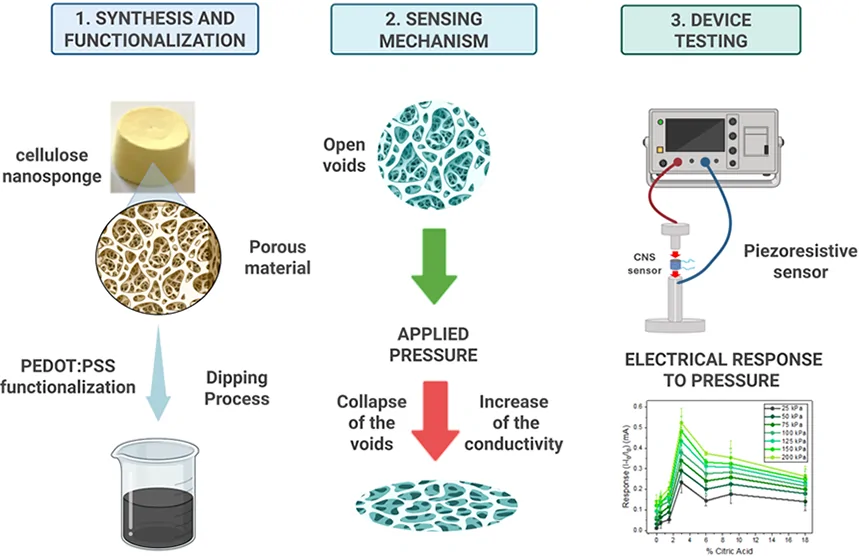

## 1 Introduction

With the increasing demand for flexible wearable devices, pressure sensors have become more important. To enable widespread adoption, they must combine high performance with compatibility with existing industrial materials and reduce production costs. Additionally, pressure sensors support real-time monitoring and enable integration with IoT platforms and AI for intelligent data analysis.^1–4^

Piezoresistive sensors have attracted significant research interest due to their low cost and ease of fabrication.^5,6^ Among the various material classes, foams and soft materials are gaining particular attention as scaffolds for integrated sensors owing to their widespread industrial applications. The working principle of piezoresistive sensors relies on the deformation of the internal structure of a conductive material under an applied pressure. This deformation alters the contact state of the material, which is translated into a measurable change in electrical resistance. To impart conductivity to polymeric foams, various functionalization strategies were developed. For instance, one-dimensional materials such as silver nanowires or nanofibers^7,8^ and carbon nanotubes^9^ can be used to achieve the desired conductivity through functionalization.

Two-dimensional materials can also be employed to develop pressure sensors. Significant examples include MXene,^10^ graphene,^11^ and MoS_2_ mixed with hydroxyethyl cellulose and polyurethane.^1^

Moreover, conductive polymers, such as poly­(3,4-ethylenedioxythiophene):polystyrenesulfonate (PEDOT:PSS) combined with single-walled carbon nanotubes,^12^ polypyrrole,^13^ and polyaniline,^14^ have also been used to fabricate three-dimensional aerogels for pressure-sensing applications.^15^ PEDOT:PSS (Scheme S1) is a widely studied conductive polymer known for its excellent electrical conductivity and mechanical flexibility. These properties make it highly suitable for various ranges of applications, including organic electronics, sensors, and energy-storage devices. PEDOT:PSS can be easily processed in aqueous solutions, enabling straightforward fabrication techniques such as spin coating, printing, and dipping.^16–18^

Sustainability has become increasingly important in sensor development. As an abundant, renewable, and recyclable material, cellulose is a promising template for sustainable sensors. Its tunable surface chemistry also enables functionalization strategies to improve the conductivity and sensitivity.^19–23^

For this reason, in the realm of integrated pressure sensors, extensive research has been conducted using cellulose-based materials.^1,6,24–29^ These latter offer several advantages, including lightweight properties, high porosity, and excellent mechanical flexibility, which make them well-suited for sensitive pressure detection.

Considering the literature on cellulose-based piezoresistive sensors, most studies employ graphene, polyaniline, or MXene as conductive components,^30^ combined with cellulose nanofibrils (CNFs) or cellulose nanocrystals (CNCs) to fabricate sensing aerogels capable of monitoring bending, blinking, or pulse signals. More rarely, the devices are employed to build pressure sensors; one of these examples is reported by Wang et al.,^31^ who developed a device to monitor the gait during a walk and a run. In this case, they used graphene oxide and 2,2,6,6-tetramethylpiperidine-1-oxyl (TEMPO)-oxidized cellulose nanofiber (TOCNF) to prepare piezoresistive carbon aerogel sensors comparable, in terms of dimension and application, to those presented in this study. Other interesting examples reported in the literature include mixtures of CNF and PEDOT:PSS with further addition of different cross-linking agents, such as glutaraldehyde (GA),^24^ glycidoxypropyl trimethoxysilane (GOPS),^32^ or polyethylenimine (PEI).^33^ These agents, which can be inserted directly into the PEDOT:PSS solution during the CNF synthesis process, or by dip coating, are used to enhance both the conductivity and the mechanical properties of the aerogels. Cheng et al.^33^ demonstrated that the presence of GA enhances the sensitivity of the CNF/PEDOT:PSS pressure sensor by improving its electrical conductivity and modifying its microstructure. GA acts as a dopant, promoting better charge mobility in PEDOT:PSS and facilitating stronger interactions with cellulose nanofibers and thus increasing the overall sensing performance. However, the use of GA raises concerns due to its toxicity, which may limit its applicability in sustainable and large-scale sensor fabrication.

In this work, we considered a novel class of cellulose-based nanostructured materials (cellulose nanosponges, CNSs) as suitable templates for piezoresistive sensors. CNSs were obtained by combining aqueous dispersions of TOCNF^34,35^ with branched polyethylenimine (bPEI),^36^ via a two-step protocol consisting of freeze-drying followed by thermal treatment. The latter promotes the formation of amidic bonds between the primary amines of bPEI and the carboxylic moieties present on the surface of the TOCNF. The mass ratio between TOCNF and bPEI was optimized to 1:2 to guarantee mechanical stability of the final materials.^36–38^ The resulting CNS exhibits microdimensional porosity introduced by the freeze-drying process and nanodimensional porosity as a result of nanofiber cross-linking.^39^ A key feature of this class of materials is the tunability of their properties through modifications of the synthetic protocol, making CNS a versatile platform for a wide range of applications. For instance, the addition of citric acid (CA) can enhance the mechanical and chemical stability of CNSs,^40^ while eco-design considerations, including ecotoxicological evaluation, ensure the environmental safety of the final materials.^41–43^ The process visualization for the synthesis of CNS is reported in Scheme 1. These engineered nanomaterials have been primarily developed as sorbents for water decontamination from heavy metals and organic dyes.^34,41,44^ However, their peculiar structure and the tunability of their properties based on the formulation make them excellent candidates for piezoresistive sensing applications.

**Scheme 1:**
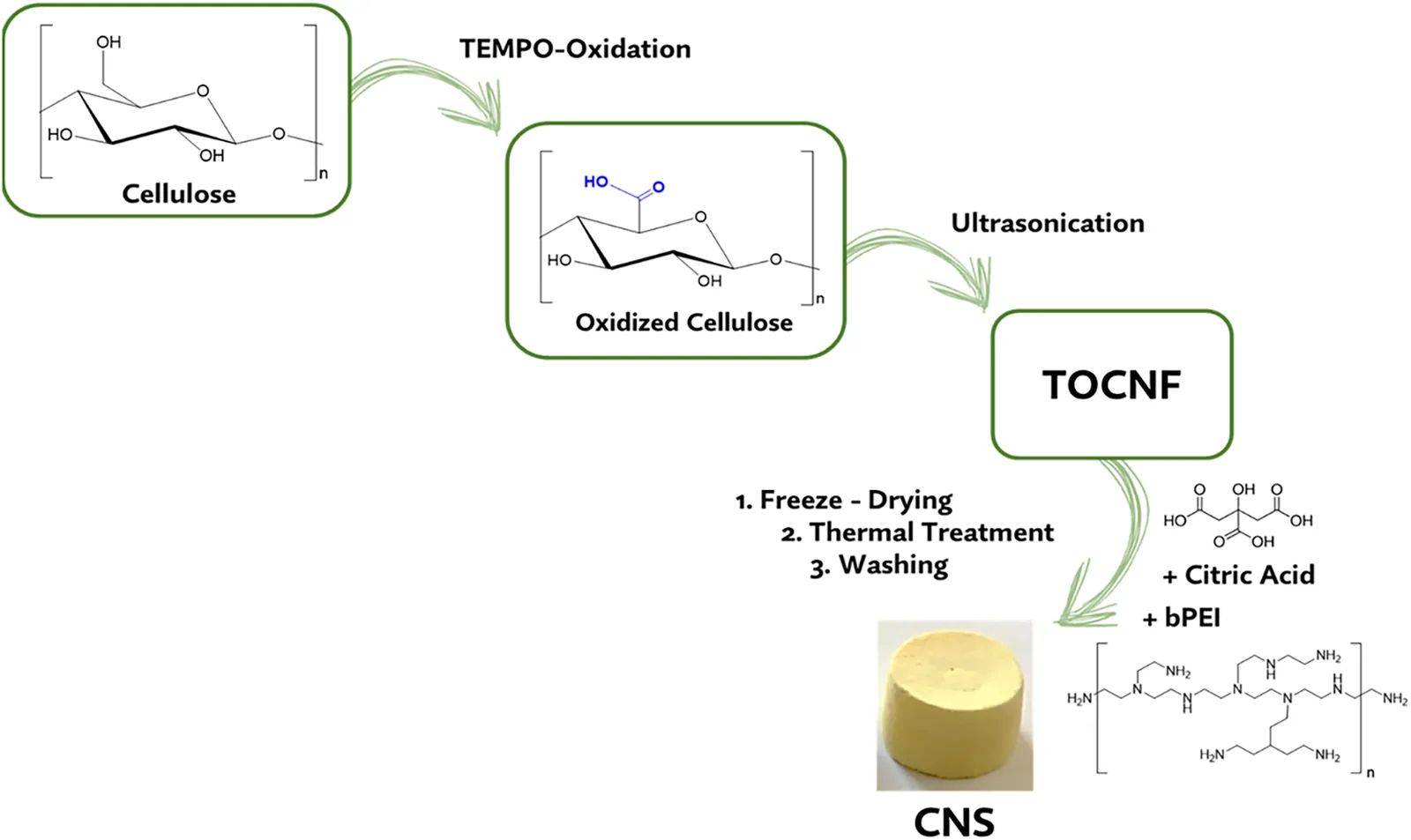
Overview for the Synthesis of CNS

Building on this potential, herein, we report the development of a new family of integrated piezoresistive sensors obtained by coating CNS scaffolds with the conductive PEDOT:PSS polymer. The study focuses on the performance of the sensors, with particular emphasis on how varying the CA content influences their piezoresistive responses.

## 2 Materials

Reagents were purchased from Merck KGaA (Darmstadt, Germany) unless otherwise specified. Cotton linter (99% purity) was provided by the Bartoli Spa paper mill (Capannori, Italy). Deionized water was produced with a Millipore Elix Deionizer with Progard S2 ion exchange resins.

### 2.1 Synthesis of CNS

CNS were synthesized using a refined protocol based on Melone et al.^36^ and Fiorati et al.^40^ The equipment used in the procedures includes a Branson Sonifier 250 equipped with a 6.5 mm probe tip, an Argo Lab TCF50 laboratory oven (Giorgio Bormac S.r.l., Carpi, Italy), a magnetic stirrer (microstirrer, VELP Scientifica, Italy), an SP Scientific BenchTop Pro Lyophilizer (Perugia, Italy), and a laboratory stove (Mazzali, Monza, Italy). TEMPO-oxidized cellulose (TOC) was derived from cotton linter through the regioselective conversion of the C6 hydroxyl groups of glucopyranose units into carboxylic acids using a TEMPO/KBr/NaClO system. Ultrasonication of TOC promoted the cleavage of the hierarchical structure of oxidized fibers, leading to the formation of TOCNF. A 3% (w/w) aqueous suspension of TOCNF was combined with 25 kDa bPEI (bPEI/TOCNF 2:1 weight ratio) and CA (variable amounts according to Table 1) to form a stable and homogeneous dispersion. This mixture was subsequently frozen at −20 °C, freeze-dried, and thermally treated at 98 °C for 16 h to produce the final cross-linked material (Scheme 1).

**Table 1:** List of Formulations Used in the Study^a^

Entry	Name	CA [%mmol]	PEDOT:PSS [dips]
1	*CNS0_0*	0	0
2	*CNS0_0.5*	0.5	0
3	*CNS0_1.5*	1.5	0
4	*CNS0_2*	2	0
5	*CNS0_3*	3	0
6	*CNS0_9*	9	0
7	*CNS1_0*	0	1
8	*CNS1_0.5*	0.5	1
9	*CNS1_1.5*	1.5	1
10	*CNS1_2*	2	1
11	*CNS1_3*	3	1
12	*CNS1_9*	9	1
13	*CNS2_0*	0	2
14	*CNS2_0.5*	0.5	2
15	*CNS2_1.5*	1.5	2
16	*CNS2_2*	2	2
17	*CNS2_3*	3	2
18	*CNS2_9*	9	2
19	*CNS2_18*	18	2

a The percentage of CA is related to the mmol of primary amino groups of bPEI, while the ratio between TOCNF and bPEI is expressed in mass equivalents and maintained constant (1:2). The column related to PEDOT:PSS expresses the number of dips undergone by CNS.

### 2.2 Coating of CNS with PEDOT:PSS

CNS were functionalized with a PEDOT:PSS 1000 solution containing 2% ethylene glycol and 1% DBSA water solution. Specifically, 100 g of PEDOT:PSS solution was mixed with 2 g of ethylene glycol and 1 g of DBSA. For the initial functionalization, the samples were dipped for 2 min into separate beakers containing the prepared solution, and then dried in a laboratory oven at 80 °C for 1 h (Scheme S1). The second functionalization included the same steps as the first functionalization, followed by an additional dipping of the CNS samples in the same PEDOT:PSS solution for 1 min and a subsequent drying step in the laboratory oven at 80 °C for 1 h. Table 1 summarizes all the formulations used in the tests.

### 2.3 Pressure Tests

The functionalized CNS was tested with a pressure application device coupled with a high-resolution electrometer (Agilent B2987A). The pressure application device was a piston built of Teflon with a diameter of 1.8 cm and length of 10 cm. The setup is configured to apply pressure in the range of 25–200 kPa to the sample by adding different weights over the Teflon piston (the relative pressures were calculated by dividing the applied force expressed in Newtons by the area of the circular piston). The sequence of increasing weight (0.5, 1, 1.5, 2, 2.5, 3, 4, and 5 kg) was applied to all the CNS previously reported in Table 1. The contacts were made with two copper plates (1.8 cm in diameter) attached to the two sides of the device. The measurements were made by applying a voltage of 0.5 V and each weight for 5 s, followed by 5 s of release before the subsequent heavier weight application. All of the samples have been tested in triplicate, and the reported measures were the mean values. The error bars were calculated by dividing the deviation standard for the square root of the number of tests. To assess the time-dependent stability of CNS2_3, pressure was monitored for 3 days under applied loads of 0.5, 1, 1.5, 2, 2.5, 3, 4, and 5 kg.

### 2.4 Morphological and Chemical Characterization

Scanning electron microscopy (SEM) analyses were performed with an FESEM/FIB ZEISS Auriga Compact instrument, using a secondary electron (SE2) detector, EHT 3 kV, working distance between 5 and 8 mm, and two fixed magnitude values (374× and 1100×). Samples were prepared by putting small pieces of sliced CNS on carbon tapes and covering them with gold under vacuum for about 30 s (performed with an Edward Scancoat Pirani 501).

The FTIR-ATR spectra of the samples were recorded using a Nicolet iS50 spectrometer (Thermo Fisher Scientific S.p.A.) equipped with a single-bounce ZnSe ATR accessory. For each sample, 64 scans were collected over the 650–4000 cm^–1^ spectral range. Data were plotted using OriginPRO 2022 software, version 9.9.0.220.

The elemental analyses of CNS sponges were obtained using a Costech ECS 4010 analyzer.

## 3 Results and Discussion

CNS were first synthesized according to the standard procedure, with different percentages of CA, providing a first family of scaffolds (CNS0_, Table 1, entries 1–6) to investigate the potential role of this cocross-linker in final pressure sensor performance after PEDOT:PSS coating. The first set of sensors was prepared by one dip into the PEDOT:PSS solution, following Scheme 1, obtaining the set of sensors CNS1_ (Table 1, entries 7–12).

The second set of sensors, CNS2_ (Table 1, entries 13–19), were prepared by double dipping the samples in the PEDOT:PSS solution as described in Section 2.2 and in Scheme S1.

Figure S1 in SI reports the amount of PEDOT:PSS sorbed after the first dipping step, after the second dipping step, and after the combined functionalization (first plus second steps). The values for the first step and for the total sorption after the two steps were obtained experimentally. In contrast, the sorbed amount during the second functionalization was determined indirectly by subtracting the first-step value from the total. All sorption values were evaluated gravimetrically after a drying process performed to remove residual water (Table 2 and Figure S1 in SI). Overall, a clear increasing trend in PEDOT:PSS uptake is observed after each successive functionalization step. For all samples, the second functionalization significantly enhances polymer uptake compared to the first treatment alone, and the combined treatment (first + second functionalization) results in the highest absorption values. The first functionalization yields relatively low absorption values (≈6–8%) across all samples, whereas a marked increase is observed after the second functionalization. In this case, the sorption trend with respect to CNS composition is nonlinear. From CNS_0 to CNS_1.5, a progressive increase in absorbed PEDOT:PSS is observed, with CNS_1.5 showing the highest uptake under all functionalization conditions (≈38–40% for combined treatment). A gradual decrease is then observed from CNS_3 to CNS_9. For CNS_18, the absorption increases again but does not reach the maximum observed at CNS_1.5.

**Table 2:** Percentage of PEDOT:PSS 1000 Only Absorbed for Each Sensor When Double Functionalized^a^

Sensor	% adsorbed PEDOT–first dipping	% adsorbed PEDOT–second dipping	% absorbed PEDOT–sum first and second dipping
*CNS0_0*	5.99	7.54	13.54
*CNS0_0.5*	7.95	20.32	28.27
*CNS0_1.5*	7.72	30.85	38.57
*CNS0_3*	6.84	19.00	25.84
*CNS0_6*	7.44	12.42	19.86
*CNS0_9*	8.24	9.41	17.65
*CNS0_18*	6.36	22.42	28.78

a Both the first and second absorptions are depicted.

Elemental analysis was performed on CNS0_3, CNS1_3, and CNS2_3 samples (Table S1), as these formulations exhibited the best pressure-sensing performance, in order to evaluate the effective distribution of PEDOT:PSS within the CNS materials. The results show an increase in the amount of PEDOT:PSS deposited on the sponge after the two impregnation steps, which can be inferred from the decrease in the nitrogen percentage since PEDOT:PSS does not contain nitrogen atoms.

Considering the CNS formulations containing 3%_mmol_ of CA, Table S1 shows that the relative nitrogen content decreases from 14.18% to 11.87% after the first dipping, and further decreases to 9.05% after the second impregnation.

Still focusing on CNS with 3%_mmol_ CA, we exploited FTIR spectroscopy to further characterize the obtained materials for a better comprehension of final performances. FTIR evidenced a progressive decrease in signals related to the functional groups specific to the CNS.

FTIR analysis (Figure S2 in SI) revealed a progressive decrease in the signals associated with CNS-specific functional groups, accompanied by a marked increase in the bands attributed to the PEDOT:PSS coating. Spectra acquired on the outer surface of the material showed more intense PEDOT:PSS-related signals, whereas spectra collected from the bulk exhibited a lower contribution to the coating. This trend was observed for both single- and double-coating applications; however, in the latter case, a more homogeneous distribution of the coating was suggested. This heterogeneity was also visually apparent as the external surface of the material appeared darker than the internal region.

The FTIR spectrum of CNS0_3, extensively described in the literature,^45,46^ exhibited characteristic bands in the 900–1400 cm^–1^ region, which were attributed to the stretching and bending vibrations of C–C, C–O, −CH, and −CH_2_ groups in cellulose. Bands observed at approximately 1588 cm^–1^ and 1650 cm^–1^ were respectively associated with residual – COO^–^ groups of TOCNF and with C=O stretching of amide bonds formed during the thermal cross-linking process. The broad band centered at around 3340 cm^–1^ was assigned to OH and NH stretching modes present in both TOCNF and bPEI.

The presence of the PEDOT:PSS coating was clearly evidenced by the bands at approximately 1006, 1032, 1123, 1187, and 1510 cm^–1^, whose intensity increased with the number of dipping cycles and resulted more pronounced on the external surface of the material. This effect was further highlighted by comparing the PEDOT:PSS-related bands at ∼1006 and 1032 cm^–1^ with the TOCNF-associated bands at 1055 and 1033 cm^–1^. Notably, the OH/NH stretching band exhibited a blue shift from ∼3340 cm^–1^ to ∼3420 cm^–1^, suggesting a rearrangement of the hydrogen-bonding network induced by interactions between the coating and the CNS matrix.

To assess potential changes in the material porosity, SEM images were acquired before and after the functionalization. SEM analysis was performed to verify the formation of a homogeneous interconnected porous network, which is essential for ensuring a reversible deformation and stable conductive pathways during repeated compression. For freeze-dried cellulose aerogels, pore sizes ranging from tens to several hundred micrometers are generally considered suitable for pressure sensing. SEM images of CNS0_3 and CNS2_3 are reported in Figure S3 in the Supporting Information. As observed from the SEM analysis, the microporous structure of the CNS remained preserved even after coating with the conductive polymer PEDOT:PSS with no evidence of pore blocking.

At this stage, the CNS samples were tested as pressure sensors by applying different weights and measuring the resulting conductivity. Figure 1a shows the responses of the CNS1 series samples at different pressures for each tested sensor and functionalization. In all cases, the response remained proportional within the analyzed pressure ranges. This linear relationship represents an important feature for sensor application, as it allows a direct correlation between the applied and the measured signal; depending on the intended application, reported sensitivities generally range from approximately 10^–2^ to 10^1^ kPa^–1^ (Table 3 and Table 4).

**Figure 1:**
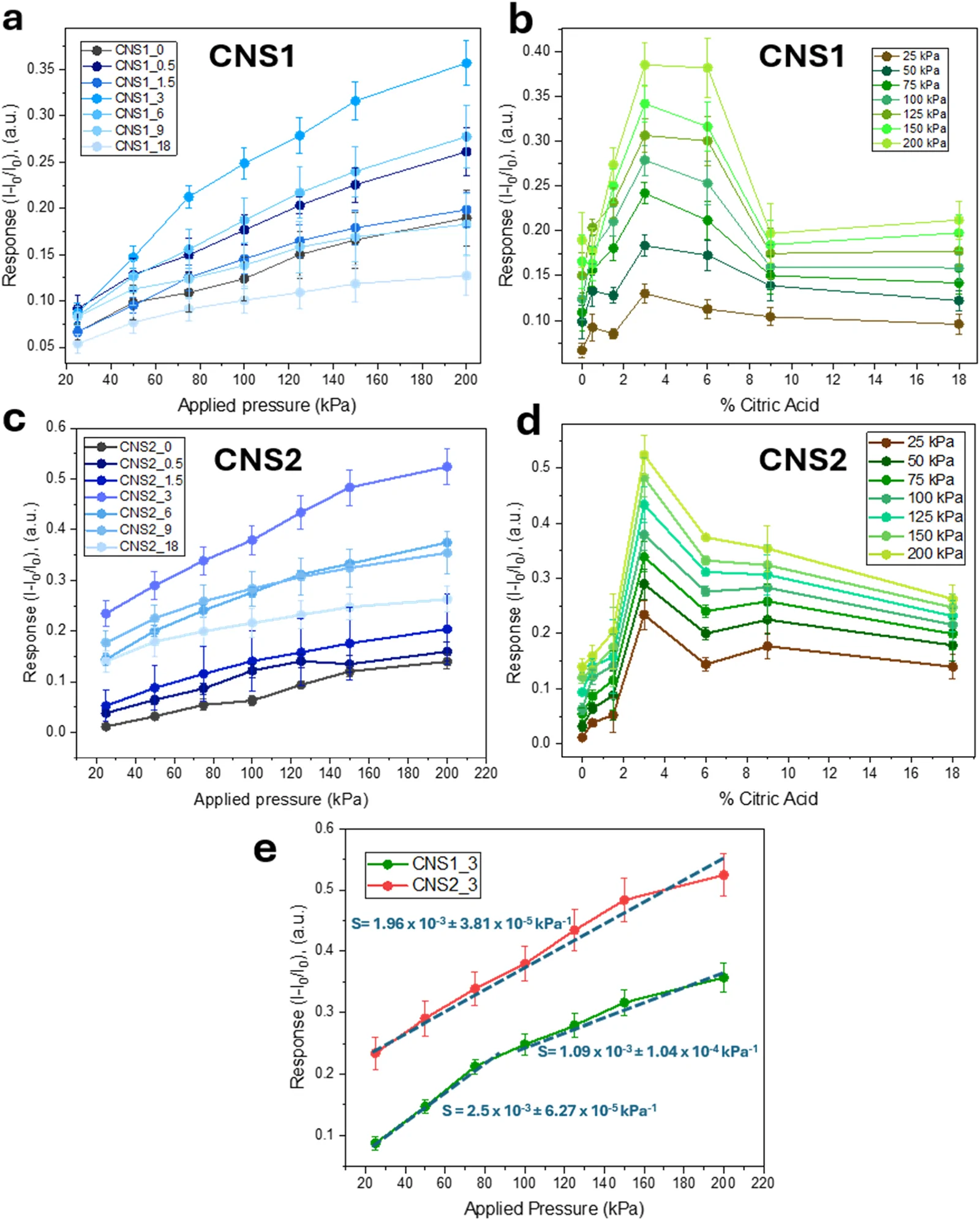
Response of sensors functionalized with PEDOT:PSS 1000 solution. (a) Response vs applied pressure for the CNS1 sample series; (b) response vs percentage of citric acid for the CNS1 sample series at different applied pressures; (c) response vs applied pressure for the CNS2 samples series; (d) response vs percentage of citric acid for the CNS2 samples series at different applied pressures; (e) comparison between the response of CNS1_3 and CNS2_3.

**Table 3:** Sensitivity and *R* ^2^ Values of the Sensors CNS1 Functionalized One Time with PEDOT:PSS 1000 Solution

Sensor	*R* ^2^	Sensitivity (kPa^–1^)
*CNS1_0*	0.981	7.53 × 10^–4^ ± 2.29 × 10^–5^
*CNS1_0.5*	0.989	1.02 × 10^–3^ ± 2.40 × 10^–5^
*CNS1_1.5*	0.994	7.19 × 10^–4^ ± 3.94 × 10^–5^
*CNS1_3*	0.982	S = 2.5 × 10^–3^ ± 6.27 × 10^–5^ kPa^–1^ (from 20 to 75 kPa) 1.09 × 10^–3^ ± 1.04 × 10^–4^ (from 100 to 200 kPa)
*CNS1_6*	0.997	1.15 × 10^–3^ ± 3.35 × 10^–5^
*CNS1_9*	0.989	6.20 × 10^–4^ ± 4.62 × 10^–5^
*CNS1_18*	0.987	4.04 × 10^–4^ ± 2.64 × 10^–5^

**Table 4:** Sensitivity and *R* ^2^ Values of the Sensors CNS2 Double Functionalized with PEDOT:PSS 1000 Solution

Sensor	*R* ^2^	Sensitivity (kPa^–1^)
*CNS2_0*	0.902	7.91 × 10^–4^ ± 2.60 × 10^–4^
*CNS2_0.5*	0.986	1.06 × 10^–3^ ± 8.61 × 10^–5^
*CNS2_1.5*	0.971	9.15 × 10^–4^ ± 3.50 × 10^–5^
*CNS2_3*	0.998	1.96 × 10^–3^ ± 3.81 × 10^–5^
*CNS2_6*	0.989	1.64 × 10^–3^ ± 9.69 × 10^–5^
*CNS2_9*	0.986	9.86 × 10^–3^ ± 6.21 × 10^–5^
*CNS2_18*	0.969	5.64 × 10^–4^ ± 5.00 × 10^–5^

These preliminary results confirmed the assumption that the CNS could serve as an effective template for piezoresponsive sensors. Upon compression, the porous structure collapses, bringing the PEDOT:PSS-coated conductive pathways closer to each other. This reduces the electrical resistance and increases the conductivity. This results in a decrease in the electrical resistance and a corresponding increase in conductivity. More specifically, the highest responses at all tested pressures were measured for CNS1_3, containing 3% CA. In contrast, both lower and higher percentages of the co-cross-linker led to reduced conductivity, with the lowest values observed for CNS1_1.5. This trend is more clearly highlighted in Figure 1b, which reports the response as a function of CA content at each applied pressure. Once again, the maximum conductivity was obtained for CNS1_3, increasing from 0.09 mA to 0.35 mA as the applied pressure increased from 25 to 200 kPa. This range of pressure was selected as these sensors could be suitable to detect medium-low pressures, for example, the pressure generated by the grasping of an object with the hand, sitting on a sensorized seat, and interface pressure in prosthetics and orthotics.

The performance of a piezoresistive sensor is primarily determined by its sensitivity, which describes the variation in the electrical resistance induced by an applied mechanical stress or pressure. This parameter is evaluated as the slope obtained from the linear fit of the sensor’s response curve. Sensitivity quantifies how strongly a sensor responds to a given stimulus and is, therefore, crucial for accurate measurements. Higher sensitivity enables the effective detection of smaller pressure changes. In our case, CNS1_0.5, CNS1_3, and CNS1_6 exhibited the highest sensitivities, with values of 1.02 × 10^–3^, 1.09 × 10^–3^, 2.5 × 10^–3^, and 1.15 × 10^–3^ kPa^–1^, respectively, with two ranges of sensitivity detected for CNS1_3 (Table 3).

On the basis of this set of results, the highest sensor response observed for CNS1_3 can be explained by considering the role of CA in the formation of the template. As previously reported, CA contributes to the cross-linking of the network by more effectively anchoring bPEI within the aerogel structure.^40^ As a consequence, an increase in the amount of CA leads to a higher level of incorporation of bPEI in the final system and to greater connectivity within the framework. While this effect enhances the mechanical strength of the material, it also progressively reduces its elasticity, which is a key parameter when the sponge is used as a template for pressure sensors. At low CA contents, CNSs are brittle, exhibiting poor mechanical resistance and therefore unsuitable behavior for pressure-sensing applications. Conversely, when excessive amounts of CA are used, CNS becomes highly rigid, leading to reduced conductivity, as observed for CNS1_9 and even more for CNS1_18.

To further investigate the potential of CNS-PEDOT:PSS systems as biobased pressure sensors, a second set of samples (CNS2_ samples, Table 1, entries 13–19) were prepared by dipping the CNS twice into the PEDOT:PSS solution, following the procedure described in Section 2.2.

The double-functionalized CNS2 samples showed a similar trend to the CNS1 samples, with the highest response values obtained for CNS2_3, moving from 0.24 mA to 0.5 mA (Figure 1c). This trend was further confirmed by the decrease in response observed for all samples containing CA amounts higher than 3%. Regarding sensitivity, CNS2_3, CNS2_6, and CNS2_9 exhibited the highest values, with sensitivities of 1.96 × 10^–3^, 1.64 × 10^–3^, and 9.86 × 10^–3^ kPa^–1^, respectively, within the linear response region (Table 4).

To evaluate the long-term durability of CNS2_3, pressure tests were conducted over a period of 3 days. The corresponding results are reported in Figure S4 of the Supporting Information. Measurements performed in the range between 25 and 200 kPa showed similar trends and comparable values throughout the tested period, indicating a stable sensor performance.

The behavior of the CNS samples can be interpreted by analyzing the FTIR data. Consistent with literature reports,^40^ higher levels of functionalization lead to the formation of weaker bonds and to an increase in the elastic modulus. In particular, a Young’s modulus nearly three times higher was observed for samples containing 18% CA compared with those containing 0%, 3%, or 9% CA, indicating a marked increase in structural stiffness and corresponding reduction in flexibility.

To assess the long-term stability of the sensor, loading/unloading cycles were performed. A stable and reproducible resistance variation under repeated loading and unloading cycles is considered a key requirement for wearable pressure sensors. On the CNS2_3 sample, repeated application-removal cycles were performed by applying a pressure of 75 kPa for 1 s and releasing for 2 s for 500 cycles (Figure 2), indicating a good preservation of the sample during all the cycles.

**Figure 2:**
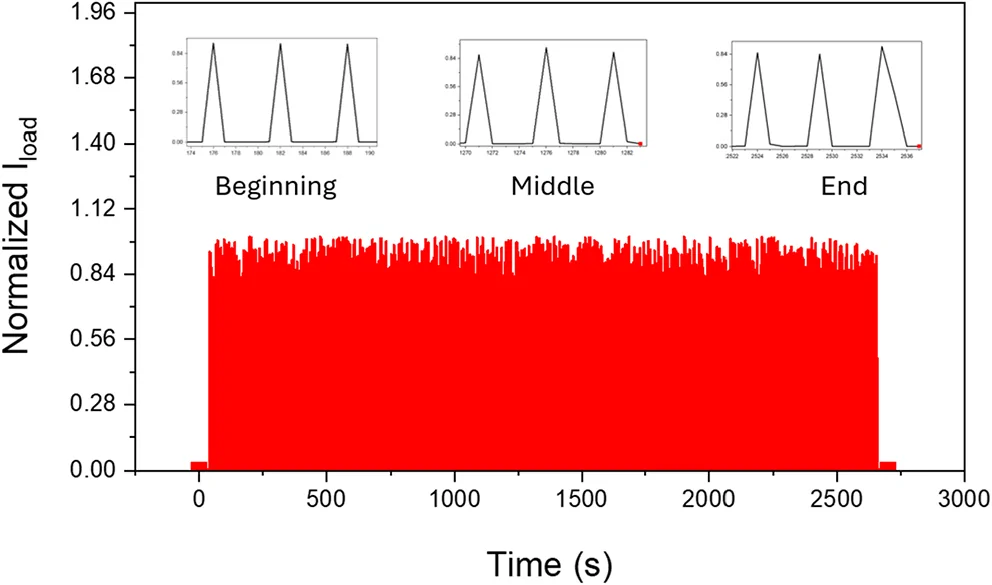
loading/unloading cycles performed by applying 75 kPa for 500 cycles.

In addition, the sensor dynamic response was analyzed by processing time-series data from step-change experiments; the rise time (10–90%) and response time were determined by identifying the signal’s steady-state value, computing 10% and 90% threshold levels, and extracting the corresponding crossing times using linear interpolation between adjacent data points. The results show an average rise time (10–90%) of approximately 1.32 s and an average response time of about 1.30 s. The close agreement between these two parameters suggests predominantly first-order behavior with a single dominant time constant. Such a response indicates that the sensing mechanism is mainly governed by the viscoelastic deformation of the 3% CA sponge structure under an applied pressure of 75 kPa, making it suitable for slow to moderate pressure monitoring applications.

Similar cellulose-based pressure sensors have been reported in the literature, such as the electrostatically assembled system developed by Cheng and co-workers,^33^ consisting of a cellulose-based pressure sensor cross-linked with PEI and PEDOT:PSS. In that work, the device was tested over a pressure range from 0 to 115 kPa and exhibited a resistance variation from approximately 175 to 110 kΩ under compression. In comparison, the present sensors display substantially lower absolute resistance values, with the best-performing samples (CNS1_3 and CNS2_3) exhibiting resistances of approximately 2 kΩ and 1.25 kΩ, respectively, under comparable operating conditions, thus indicating a more conductive percolative network.

At an applied bias of 0.5 V, the present sensor exhibits a current sensitivity of 1.96 × 10^–6^ A/kPa. By normalizing the current response to the baseline current (*I*_0_ = 5.85 × 10^–5^ A) and assuming ohmic behavior under constant bias, this corresponds to a relative sensitivity of 3.35 × 10^–2^ kPa^–1^ (≈3.35% per kPa). Over the investigated pressure range of 25–200 kPa, this relative sensitivity results in an overall current modulation of approximately 590%.

For comparison with other piezoresistive pressure sensors reported in the literature, including highly porous PEDOT:PSS/cellulose nanofiber aerogel systems,^24^ aerogel-based sensors typically exhibit higher relative resistance sensitivities, on the order of 10–15 kPa^–1^. These higher values are generally attributed to their ultralight, highly porous, and easily compressible architectures. In contrast, the present sensor relies on a bulk three-dimensional structure and operates under compressive pressure over a substantially wider pressure range, while still achieving a large and clearly distinguishable modulation of the electrical signal at low operating voltage.

These results suggest that pressure-induced reconfiguration of the conductive network within the material provides an effective sensing mechanism, placing the present system in line with other polymer-based piezoresistive pressure sensors designed for practical operating ranges as described in Table 5.

**Table 5:** Comparison of the Performance of the Sensors Developed in This Work with Other Similar Reported in the Literature

Article ref	Structure Type	Sensitivity	Response Time	Number of Cycles	Pressure sensor range
^22^	Stretchable cellulose film	GF ≫ 1.2	0.19–0.37 s	5000 cycles	Not reported
^27^	Polyurethane sponge (foam)	0.511 kPa^–1^	25 ms	>1000 cycles	0.67–60 kPa
^28^	Wood sponge (porous structure)	5.69 kPa^–1^	∼111 ms	>500 cycles	0–30 kPa
^24^	3D porous CNF/PEDOT:PSS aerogel (GA cross-linked)	15.08 kPa^–1^ (0–2 kPa); 1.91 kPa^–1^ (3–24 kPa); 0.45 kPa^–1^ (>25 kPa)	Not reported	500 cycles	0–25 kPa
This work	CNS nanosponges	1.96 × 10–3 kPa^–1^	∼1.3 s	Stable under 500 repeated cycles	20–200 kPa

Among the tested applications, and considering the tested range of pressure, these sensors can be integrated into wearable monitoring devices. For example, by incorporating the CNS sensors into a glove and connecting them to a suitable readout electronics, they can be used to monitor object grasping and hand–object interaction forces. Other potential applications include monitoring the pressure of low-pressure bicycle tires and measuring the interface pressure exerted by medical compression bandages.

## 4 Conclusions

In conclusion, we demonstrated that nanocellulose-based materials are suitable candidates for integrated pressure sensors. CNS samples with varying CA content (0–18%) were tested after one and two functionalization steps with PEDOT:PSS to evaluate the influence of the formulation on sensor performance. Our results indicate that achieving optimal sensor response requires a balance between the stiffness of the material and the amount of PEDOT:PSS present on the CNS surface. In particular, the best-performing sensors contained 3% citric acid, for both single and double functionalization, exhibiting sensitivities of 2.5 × 10^–3^ ± 6.27 × 10^–5^ kPa^–^ in the range between 25 and 75 kPa and 1.09 × 10^–3^ ± 1.04 × 10^–4^ kPa^–1^ in the range between 100 and 200 kPa and 1.96 × 10^–3^ ± 3.81 × 10^–5^ kPa^–1^ for the double functionalization.

These findings confirm that our nanostructured cellulose-based sensors are promising piezoresistive materials, offering significant advantages due to their environmental sustainability, ecocompatibility, and good mechanical properties. Leveraging the natural abundance and biodegradability of cellulose, these sensors present an ecofriendlier alternative to conventional synthetic materials. Moreover, the inherent flexibility and lightweight nature of cellulose-based materials contribute to the versatility and comfort of wearable sensors, broadening their potential applications in environmental sensing, and smart packaging. By integrating these sensors into various devices, it could be possible to achieve high performance while minimizing the environmental impact, effectively aligning technological advancement with ecological preservation.

## Supplementary Material



## Notes

Detailed synthesis of CNS; schematic representation of the functionalization process to obtain CNS1, CNS2, and the chemical structure of the monomer of the PEDOT:PSS polymer; superimposition of FTIR spectra of CNS0_3, CNS1_3, and CNS2_3; trend of the normalized responses of CNS2_3 tested for 3 days consecutive; and results of elemental analysis performed on CNS0_3, CNS1_3, and CNS2_3, to evaluate the percentage of nitrogen (N), oxygen (O), and hydrogen (H) after several impregnation cycles (PDF)
