# Supplementary material for: Cellulose-Based Nanosponges with PEDOT:PSS as Pressure Sensors with Tunable Elasticity: A Study on Modulation of Mechanical Properties and Pressure Response
Source: ACS Omega. 2026 Oct 8;11(41):62623–31. doi: 10.1021/acsomega.6c03688 (PMC13688518; doi:10.1021/acsomega.6c03688)
Supplement: Supplementary file 1 [file ao6c03688_si_001.pdf]

Supplementary information

Cellulose-based Nanosponges with PEDOT:PSS as Pressure Sensors with Tunable Elasticity: a study on Modulation of Mechanical Properties and Pressure Response

V. Vit [¥, †], L. Riva [§, †], V. Sinisi [¥], A. Fiorati [§], A. Alam [¥], A. B. Alabi [¥], C. Punta [§], N. Coppedè [¥*]*

[¥] Institute of Materials for Electronics and Magnetism (IMEM), National Research Council (CNR), Parco Area delle Scienze 37/A, 43124 Parma, Italy.

[§] Department of Chemistry, Materials, and Chemical Engineering "G. Natta" Politecnico di Milano and INSTM Local Unit, Piazza Leonardo da Vinci 32, I-20133 Milano, Italy.

[†] These two authors contributed equally to this work

* Corresponding authors: Carlo Punta

+39 02 2399 3026

carlo.punta@polimi.it

Nicola Coppedè

+39 0521 269 217

nicola.coppede@imem.cnr.it

Detailed synthesis of CNS

In a 1 L beaker, 3.5 g of oxidized cellulose was suspended in 0.14 L deionized water, in order to obtain a 2.5% w/v solution. While mixing it, granular NaOH in a stoichiometric quantity was added to the solution to reach pH around 11 (evaluated with litmus paper), obtaining a slightly yellow viscous suspension. The beaker was placed in an ice bath, and the solution was ultrasonicated (working at 20 kHz in continuous mode with an output power 50% the nominal value (200 W)) to further promote the separation of the nanofibers, obtaining a transparent and more homogeneous suspension. The latter was then acidified with concentrated HCl and filtered on a Büchner funnel under vacuum, then washed and re-suspended. This procedure was repeated with deionized water until a neutral pH was reached. Wet cellulose was removed from the filter paper and weighted; 3.5 g was assumed to correspond to the starting cellulose, while the remaining mass represented the residual water content. The remaining water necessary to obtain a 2.8-3.0% w/v TOCNFs solution for the next phase was split up in three quotas, in order to re-suspend the cellulose and dissolve the cross-linking polymer (branched polyethylenimine, bPEI) and the co-cross linker (citric acid monohydrate, CA), in three separate batches. The amount of the co-reticulant was calculated by means of the following equation:

$$m_{CA} = x\, g_{bPEI} * 7.4 \frac{mmol_{NH_2}}{g_{bPEI}} * n \frac{mmol_{CA}}{mmol_{NH_2}} * \frac{mmol_{CA*1H_2O}}{mmol_{CA}} * 192.12 \frac{mg_{CA}}{mmol_{CA}} = y\, mg_{CA}$$

where the amount of CA (n) varies from a minimum of 0 to a maximum of 0.18.

Once dissolved in water, the bPEI and CA were slowly added to the cellulose solution, while continuously stirring until obtaining a white and homogeneous hydrogel, which was placed in 24-well well-plates (about 15.6 mm diameter, half-filled with about 2 mL each) and quickly frozen at –35 °C. After 12 hours, the well-plates were moved to the freeze-dryer, which allowed water removal by sublimation, thus retaining the internal structure of the frozen hydrogels. At the end of the process, white cylindrical-shaped spongy aerogels were obtained. They were removed from the wells and placed in the laboratory oven, at the initial temperature of 55 °C. The temperature was then slowly raised up to 98 °C in 4 hours, in order to avoid sudden overheating and prevent the risk of burning the material. Around 100 °C, the water formed due to the reticulation reactions between carboxylic moieties and amines (formation of amidic bonds) was quickly evaporated, allowing the proper reticulation of the material. CNS were removed from the oven after 16 hours. At the end of the

process, CNS were washed six times with deionized water and the last time with ethanol to eliminate the excess of bPEI.

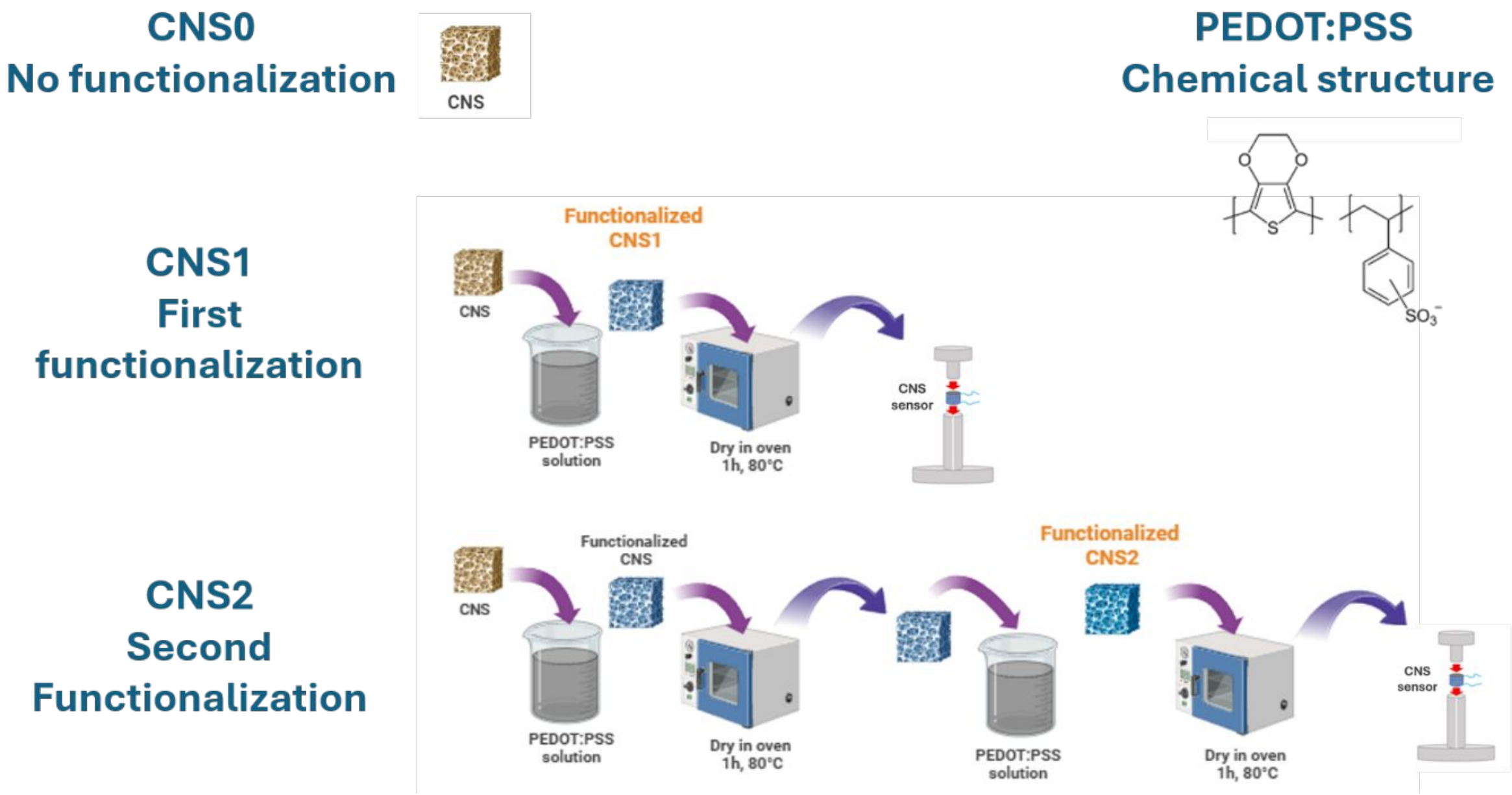

Scheme S1. Schematic representation of the functionalization process to obtain CNS1, CNS2 and the chemical structure of the monomer of the PEDOT:PSS polymer. CNS0 represents the CNS without functionalization. [Scheme S1 was created using BioRender (BioRender.com)]

Table S1. Results of elemental analysis performed on CNS0_3, CNS1_3 and CNS2_3, to evaluate the percentage of Nitrogen (N), Oxygen (O) and Hydrogen (H) after several impregnation cycles.

Sample name	N [%]	C [%]	H [%]
CNS0_3	14.18 ± 0.2	44.91 ± 0.5	7.88 ± 0.3
CNS1_3	11.87 ± 0.2	47.28 ± 0.5	8.33 ± 0.3
CNS2_3	9.05 ± 0.2	49.20 ± 0.5	8.28 ± 0.3

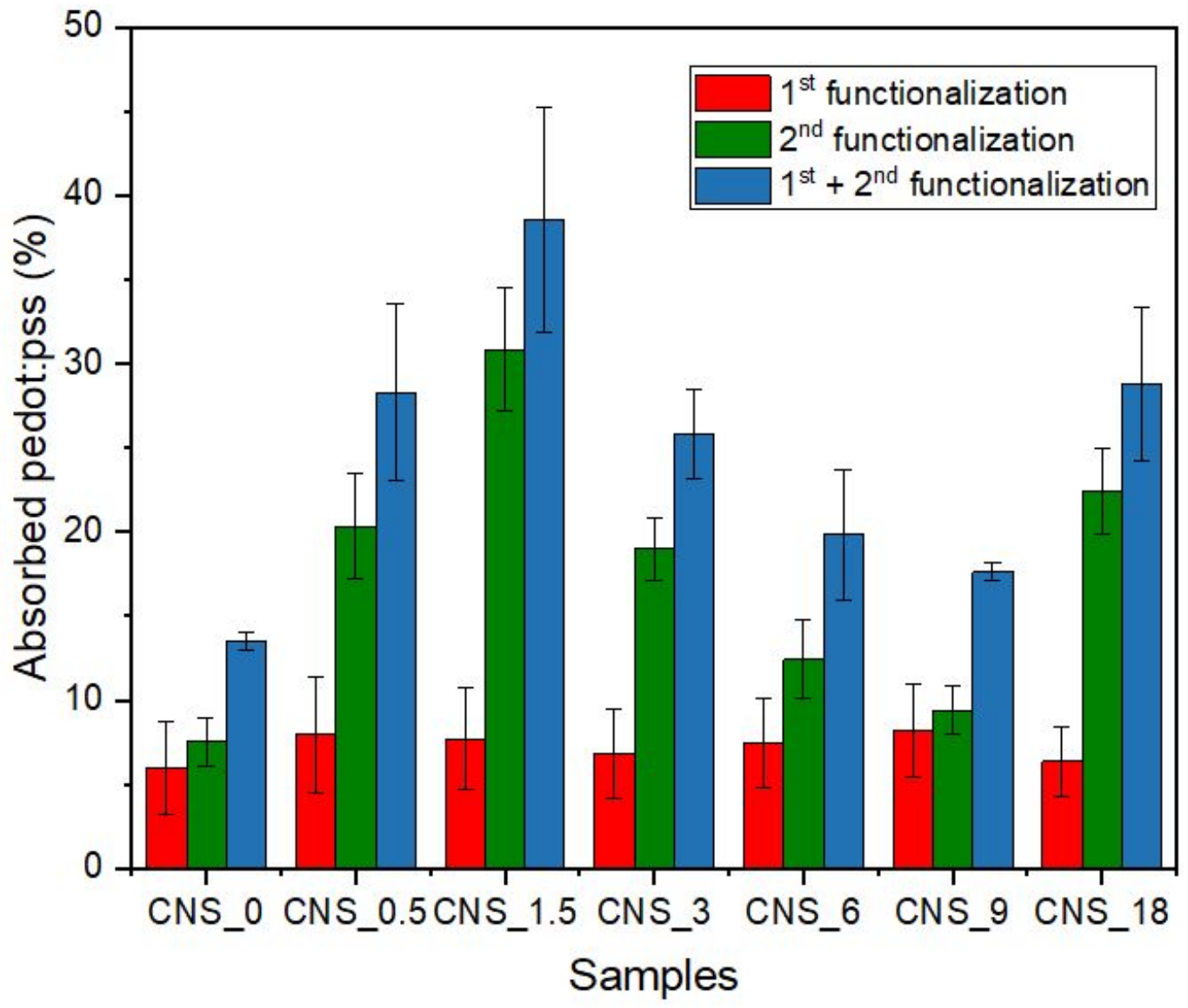

Figure S1. Comparison of percentage of absorbed PEDOT:PSS 1000 for each tested sample after the first functionalization (red), the second one (green), and the sum of the two (blue).

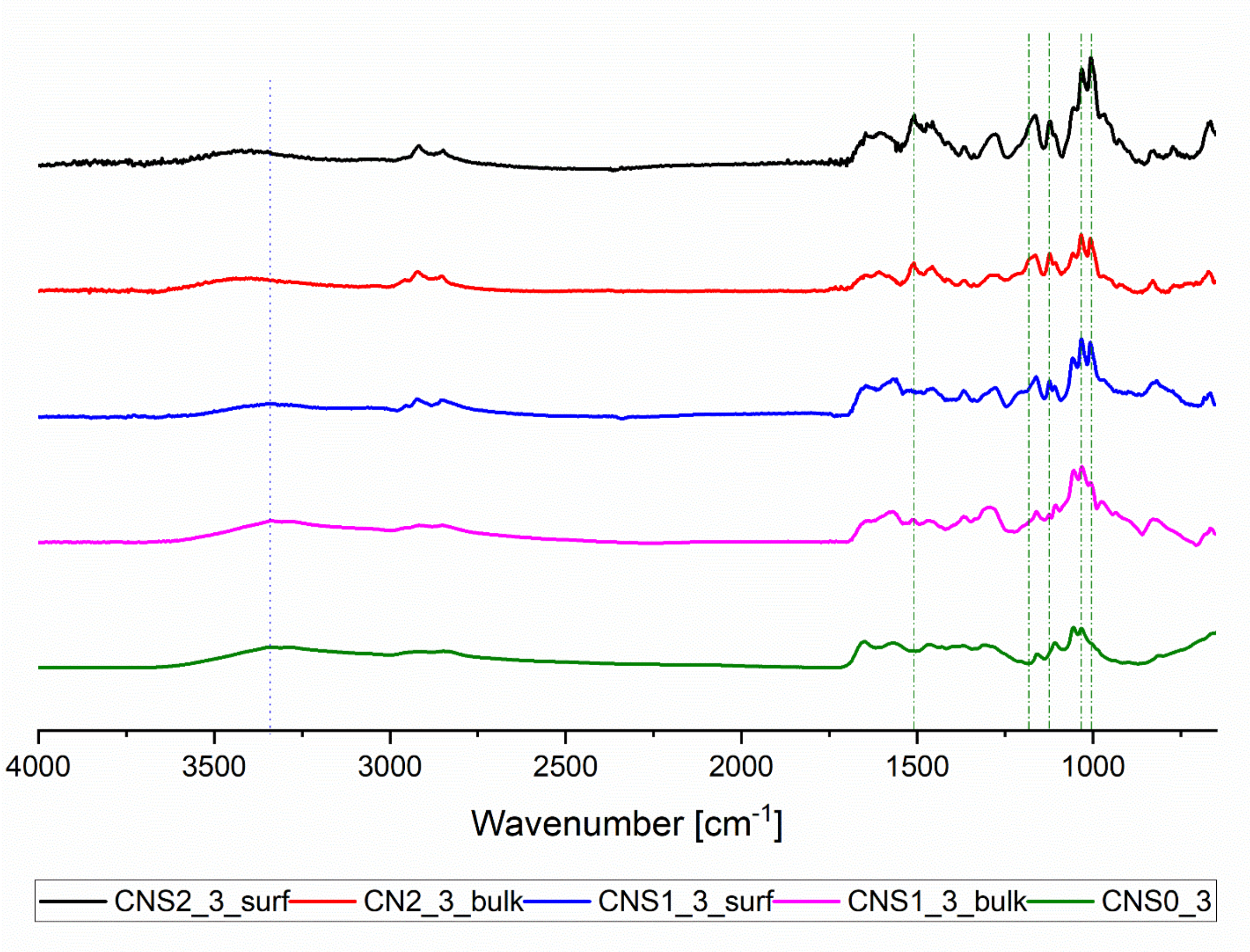

Figure S2. Superimposition of FT-IR spectra of CNS0_3, CNS1_3, and CNS2_3. The suffix surf or bulk appended to the sample code indicates measurements performed on the external surface or within the internal region of the material, respectively. The green dashed–dotted line highlights characteristic PEDOT:PSS signals, while the blue dotted line serves as a reference for the OH/NH stretching region.

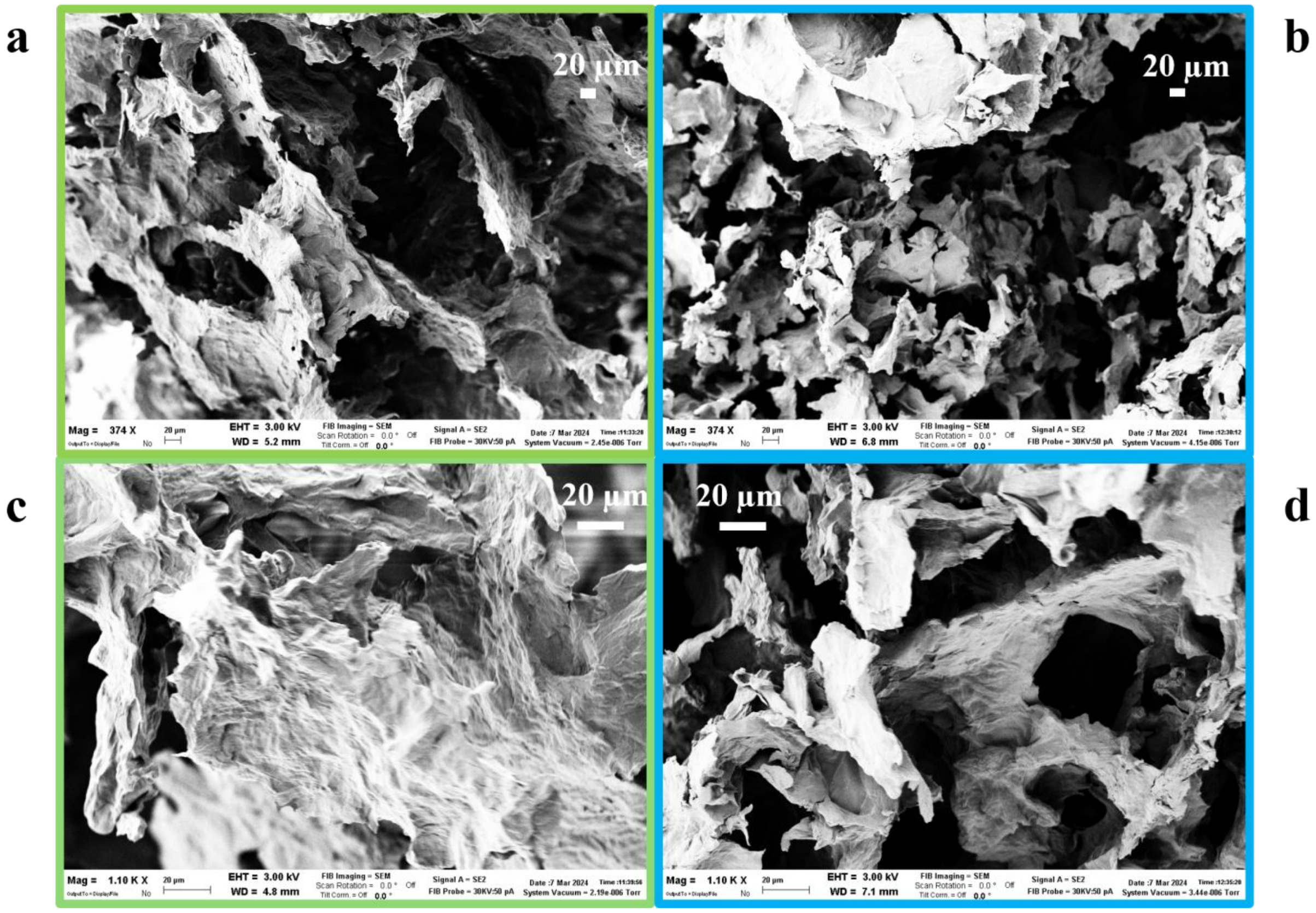

Figure S3. SEM images at 374x and 1100x MAG before (a and c respectively, CNS0_3), and after PEDOT:PSS impregnation (b and d, respectively, CNS2_3).

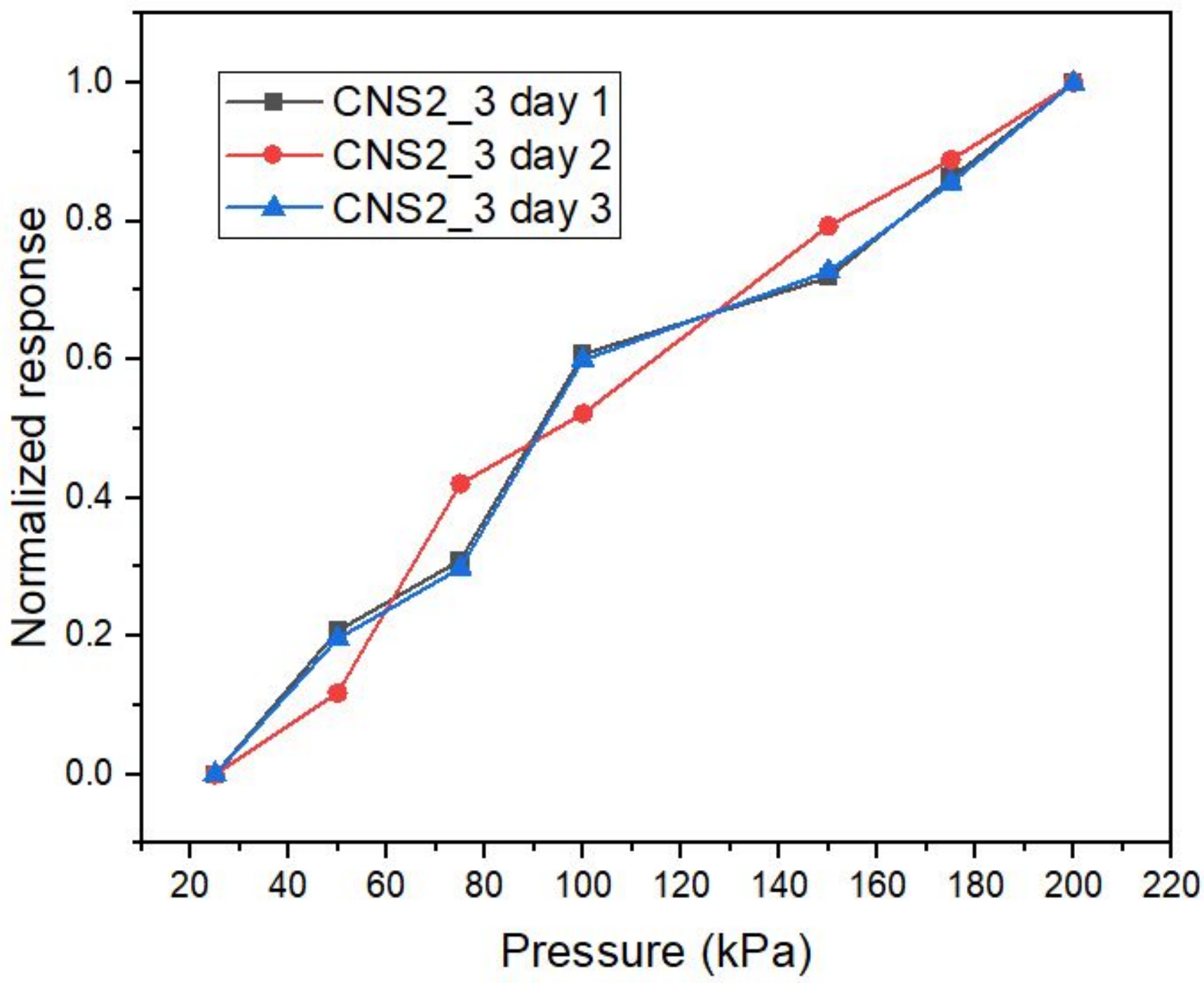

Figure S4. Trend of the normalized responses of CNS2_3 tested for 3 days consecutive.
